# Dysregulated bile acid receptor-mediated signaling and IL-17A induction are implicated in diet-associated hepatic health and cognitive function

**DOI:** 10.1186/s40364-020-00239-8

**Published:** 2020-11-06

**Authors:** Prasant Kumar Jena, Lili Sheng, Michelle Nguyen, Jacopo Di Lucente, Ying Hu, Yongchun Li, Izumi Maezawa, Lee-Way Jin, Yu-Jui Yvonne Wan

**Affiliations:** 1 Department of Medical Pathology and Laboratory Medicine, University of California, Davis Health, Room 3400B, Research Building III, 4645 2nd Ave, Sacramento, CA 95817 USA; 2 Department of Pediatrics, Cedars Sinai Medical Center, Los Angeles, CA 90048 USA; 3 Institute of Interdisciplinary Integrative Medicine Research, Shanghai University of Traditional Chinese Medicine, Shanghai, 201203 China; 4 Department of Gastroenterology, Zhujiang Hospital, Southern Medical University, Guangzhou, 510282 China; 5 Department of Infectious Diseases, Nanhai Hospital, Southern Medical University, Foshan, 528200 China

**Keywords:** Gut-liver axis, Gut-brain axis, Inflammation, Neuroplasticity, Metabolic syndrome, Gut microbiota, Bile acid receptor, Dementia, Cognition

## Abstract

**Background:**

Chronic consumption of high sugar and high fat diet associated with liver inflammation and cognitive decline. This paper tests a hypothesis that the development and resolution of diet-induced nonalcoholic fatty liver disease (NAFLD) has an impact on neuroplasticity and cognition.

**Methods:**

C57BL/6 wild-type mice were fed with either a healthy control diet (CD) or a fructose, palmitate, and cholesterol (FPC)-enriched diet since weaning. When mice were 3-months old, FPC diet-fed mice were randomly assigned to receive either FPC-enriched diet with or without 6% inulin supplementation. At 8 months of age, all three groups of mice were euthanized followed by analysis of inflammatory signaling in the liver and brain, gut microbiota, and cecal metabolites.

**Results:**

Our data showed that FPC diet intake induced hepatic steatosis and inflammation in the liver and brain along with elevated RORγ and IL-17A signaling. Accompanied by microglia activation and reduced hippocampal long-term potentiation, FPC diet intake also reduced postsynaptic density-95 and brain derived neurotrophic factor, whereas inulin supplementation prevented diet-reduced neuroplasticity and the development of NAFLD. In the gut, FPC diet increased *Coriobacteriaceae* and *Erysipelotrichaceae*, which are implicated in cholesterol metabolism, and the genus *Allobaculum,* and inulin supplementation reduced them. Furthermore, FPC diet reduced FXR and TGR5 signaling, and inulin supplementation reversed these changes. Untargeted cecal metabolomics profiling uncovered 273 metabolites, and 104 had significant changes due to FPC diet intake or inulin supplementation. Among the top 10 most affected metabolites, FPC-fed mice had marked increase of zymosterol, a cholesterol biosynthesis metabolite, and reduced 2,8-dihydroxyquinoline, which has known benefits in reducing glucose intolerance; these changes were reversible by inulin supplementation. Additionally, the abundance of *Barnesiella*, *Coprobacter*, *Clostridium XIVa*, and *Butyrivibrio* were negatively correlated with FPC diet intake and the concentration of cecal zymosterol but positively associated with inulin supplementation, suggesting their benefits.

**Conclusion:**

Taken together, the presented data suggest that diet alters the gut microbiota and their metabolites, including bile acids. This will subsequently affect IL-17A signaling, resulting in systemic impacts on both hepatic metabolism and cognitive function.

**Supplementary Information:**

The online version contains supplementary material available at 10.1186/s40364-020-00239-8.

## Introduction

Non-alcoholic fatty liver disease (NAFLD) is associated with reduced cognitive performance in adults through the impairment of psychomotor speed and learning ability, suggesting a link between hepatic metabolism and brain function [1]. Additionally, there is an increased prevalence of metabolic syndromes in patients with NAFLD, which is considered a risk of cognitive decline [2]. Moreover, early-life exposure to a Western diet (WD) impairs hippocampal-dependent memory and neuroplasticity [3]. This paper tests a hypothesis that the development and resolution of diet-induced NAFLD has an impact on brain neuroplasticity. We also studied whether highly fermentable fiber inulin can prevent WD-induced liver disease and WD-impaired neuroplasticity simultaneously.

Emerging evidence reveals the significance of diet via the gut microbiota in regulating liver health [4, 5]. The gut microbiota also affects neuronal functions by modifying the production of vitamins, neurotransmitters, and neuroactive microbial metabolites such as short-chain fatty acids (SCFA) [6]. Additionally, the gut microbiome and brain communicate in a bidirectional manner, modulating the activation of microglial cells and affecting myelination as well as neurogenesis in the brain [7, 8]. Thus, we aim to establish the gut microbiota and their associated metabolites implicated in the development of NAFLD and cognitive decline as well as their resolutions.

Gut dysbiosis alters the composition of the bile acid (BA) pool, resulting in compromised BA receptor farnesoid x receptor (FXR) and Takeda G protein-coupled receptor 5 (TGR5)-regulated signaling [9]. Both FXR and TGR5 are expressed in the liver as well as the gut, and they are detectable in the brain where they regulate metabolism and immunity [10, 11]. Activation of TGR5 with its agonist INT-777 ameliorates synaptic dysfunction against Aβ_1–42_-induced neurotoxicity [12]. BAs also regulate the gut microbiota directly via their antibacterial activity [9]. Additionally, the secondary BAs are bacterial metabolites, which are implicated in liver carcinogenesis and cognitive decline [13, 14]. Therefore, BAs and their regulated signaling explains how diet via the gut microbes affects health at the systemic level.

Inulin is a nondigestible fiber present in a wide variety of fruits and vegetables. It is commonly used as a dietary supplement for weight loss and lowering serum lipids [15]. Through gut microbial fermentation, inulin generates SCFAs that have health benefits [16]. It has been shown that inulin improves mood as well as memory and digestion in humans [17]. Inulin also ameliorates castration-induced cognitive decline [18]. Unexpectedly, although long-term (6 months) supplementation of inulin (7.5%) improves metabolic syndrome in toll-like receptor 5 (TLR5)-knockout mice, many of these mice develop icteric hepatocellular carcinoma (HCC) [19]. These surprising findings suggest that long-term consumption of inulin is a potential risk for developing liver disease; therefore, there is a need to develop precision dietary supplementation [20]. Together, this study aims to further understand the impact of inulin through altering gut microbes and metabolites, which thereby affects liver and brain health.

In contrast to our previously used WD (TD.140414) [4, 21], which has a moderate amount of fat and high sucrose, we examined the long-term (7 months) effects of another diet named “fast food diet” or fructose, palmitate, and cholesterol (FPC) diet on liver and brain health in current study. FPC diet has been used to induce severe liver fibrosis and non-alcoholic steatohepatitis (NASH) [22]; however, its effects on the brain have never been studied. Our data show that different Western diets that contain enriched sugar and fat consistently compromised neuroplasticity and induced NAFLD. However, inulin supplementation concomitantly prohibited the negative effects of those WD in the brain and liver. Furthermore, gut microbes and metabolites associated with cognitive function as well as NAFLD development or prevention have been established in this study. Our data also suggest that BA receptor-regulated signaling likely has a role in regulating both liver and brain health.

## Materials and methods

### Mice

C57BL/6 wild-type (WT) male mice that were free of specific pathogens (Jackson Laboratory, Sacramento, CA, USA) were contained in steel microisolator cages with a 12-h light/dark cycle at 22 °C. After weaning, mice were fed with either a control diet (CD) containing 6.2% fat, 44% carbohydrate, and 18% protein or an FPC diet constituting 29% fat, 34% sucrose, and 1.25% cholesterol (w/w, TD. 160,785, Envigo, Indianapolis, IN, USA) plus 42 g/L glucose and fructose (55%/45%, w/w) in drinking water. At 3 months of age, FPC diet-fed mice were randomly assigned to two subgroups with or without inulin (6%, Micro ingredients, Montclair, CA, USA) supplementation for 5 months. At 8 months of age, all three groups (*n* > 4) of mice were euthanized. Experiments were conducted in accordance with the National Institutes of Health Guidelines for the Care and Use of Laboratory Animals under protocols approved by the Institutional Animal Care and Use Committee of the University of California, Davis.

### Histopathology and biochemical analysis

Liver histology was analyzed by hematoxylin and eosin (H&E) stained section. Steatosis scores were graded on a scale of 0 (< 5%), 1 (5–33%), 2 (34–66%), and 3 (> 66%) based on the percentage of area that had fat [23]. Serum alanine aminotransferase (ALT) and alkaline phosphatase (ALP) (Pointe Scientific, Canton, MI, USA) levels were quantified per manufacturer protocols.

### Western blot analysis

Liver and brain proteins (40 μg) were subjected to SDS-PAGE followed by transferring to PVDF membranes. The following primary antibodies (dilutions) were used: FASN (1:1000; Cell Signaling Technology, Danvers, MA, USA), CD36 (1:1000; Bioss Antibodies, Woburn, MA, USA), SCD1 (1:1000; Cell Signaling, Danvers, MA, USA), IL-17A (1:3000; eBiosciences, San Diego, CA, USA), TNFα (1;1000; Bioss Antibodies, Woburn, MA, USA), FXR (1:250; Santa Cruz Biotechnology, CA, USA), HNF4α (1:1000; Aviva system biology, San Diego, CA, USA), SHP (Santacruz Biotechnology, Dallas, Texas, USA), PSD-95 (1:1000; Cell Signaling, Danvers, MA, USA), BDNF (1:1000; Millipore Sigma, St. Louis, MO, USA), TGR5 (1:3000; Lifespan Biosciences, WA, USA), and β-ACTIN (1:10000; Millipore Sigma, St. Louis, MO, USA). Horseradish peroxidase-conjugated secondary antibodies were then used to incubate the membranes. An ECL system with Pierce SuperSignal West Pico chemiluminescent substrates (Thermo Fisher Scientific, Rockford, IL, USA) was used to detect signals. Western blots protein band intensities were quantified by using ImageJ software (Version 1.53c) showed in the supplementary Figure 1.

### Gene expression profiling

TRIzol (Invitrogen, Carlsbad, CA, USA) was used to isolate hepatic and microglial RNA followed by reverse transcription to generate cDNA. qRT-PCR was utilized on an ABI 7900HT Fast real-time PCR system using Power SYBR Green PCR Master Mix (Applied Biosystems, Foster City, CA, USA). *Gapdh* mRNA level was used to normalize mRNA levels. Primers used for qRT-PCR listed in supplementary Table 1.

### Microglia isolation

Brain microglia were isolated by enzymatical digestion using a neural tissue dissociation kit (Miltenyi Biotec, San Diego, CA) followed by magnetic-activated cell sorting with anti-CD11b coated magnetic beads (Miltenyi Biotec, Auburn, CA, USA).

### Long-term potentiation (LTP) study by electrophysiological recording

Electrophysiological recordings were performed as previously described [23]. Briefly, coronal brain slices (300 μm) were prepared and transferred to artificial cerebrospinal fluid (ACSF) described earlier [24]. After subsequent incubation for at least 1 h at room temperature, hemi-slices were transferred to the recording chamber, which was perfused with standard ACSF at a constant flow rate of ~ 2 ml/min. After stimulation of the schaffer collateral afferents, recording of field excitatory post-synaptic potentials (fEPSPs) were obtained from the stratum radiatum of the Cornu Ammonis (CA1) region in the hippocampus. Borosilicate capillaries with an outer diameter of 1.5 μm (Sutter Instruments, Novato, CA, USA) and filled with 3 M NaCl (resistance, 1–2 MΩ) were used to make extracellular recording electrodes. Baseline stimulation rate was 0.05-Hz. A Multiclamp 700B amplifier (Molecular Devices, Sunnyvale, CA) was used to filter the fEPSPs at 2 kHz and digitized at 10 kHz. Collected data were analyzed with pClamp software version 10.3 (Molecular Devices, Inc., Sunnyvale, CA, USA). Slope values of fEPSPs were considered for quantitation of the responses. After 10 mins of stable baseline recording of fEPSPs was induced every 20 s, LTP was prompted by high-frequency stimulation (HFS) comprising of a single train of 100-Hz (1 s) stimulation with the same intensity and pulse duration used in sampling of baseline fEPSPs.

### Open field behavior study

Open field behavior tests for exploratory activity were recorded during the day light phase. The SmartCage™ system (AfaSci Inc., Redwood City, CA, USA) was used to monitor the spontaneous activity. Mice explored freely for 10 min and their activities were determined by infrared beam breaks (x, y, and z photo-beam break counts) monitored and analyzed by Cage Center and CageScore 2 software, respectively (AfaSci Inc., Redwood City, CA, USA).

### 16S rRNA gene sequencing

Cecal DNA was extracted using ZR Fecal DNA Miniprep Kit (Zymo Research, Irvine, CA, USA). Barcoded 16S rRNA gene amplicons of genomic DNA Illumina (San Diego, CA, USA) sequencing was performed based on published methods [25]. Variable region 4 of the 16S rRNA gene was amplified and sequenced. A custom python based dbcAmplicons (https://github.com/msettles/dbcAmplicons) was used for demultiplexing and classifying DNA sequence reads to identify and assign the reads by expected barcode in addition to primer sequences [25]. The Ribosomal Database Project Bayesian classifier was used to assign sequences to phylotypes [26]. Reads were assigned to the first Ribosomal Database Project taxonomic level with a bootstrap score ≥ 50.

### Untargeted metabolomic study

Cecal metabolites were quantified by gas chromatography time-of-flight mass spectrometry (GC-TOFMS) [27]. Acquired spectra were processed using the BinBase database, filtered, and matched against the Fiehn Mass Spectral Library of 1200 authentic metabolite spectra with retention index and mass spectrum information or against the NIST library. Pathway analyses were generated by MetaboAnalyst 3.0 [28]. Chemical similarity enrichment analysis was performed by ChemRICH [29].

### Bioinformatics and statistical analysis

Volcano plots and Spearman’s correlations were completed with R Statistical Software (The R Project for Statistical Computing; https://www.r-project.org/). Data were expressed as means ± SD. Cecal metabolomics data were analyzed with ChemRICH [29] and MetaboAnalyst method [30]. Group differences in microbiota family level were calculated by Kruskal-Wallis test. All comparisons were calculated by two-tailed Student’s *t* test or one-way ANOVA followed by Tukey’s test using GraphPad Prism 6 software (GraphPad Software, La Jolla, CA, USA). The *p*-values are adjusted for multiple comparisons using false discovery rate. A value of *p* < 0.05 was considered statistically significant.

## Results

### Inulin prevents diet induced NAFLD

**Fig. 1:**
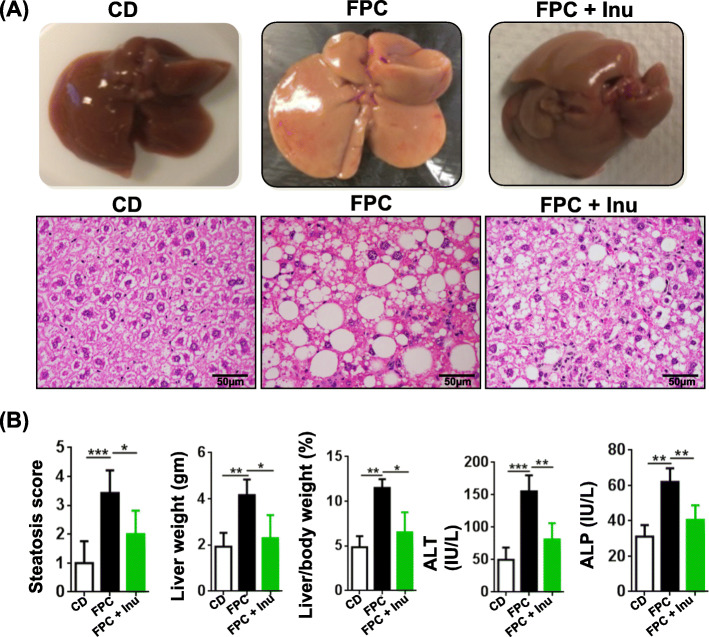
Inulin supplementation prevents diet-induced fatty liver. C57BL6/J wild-type mice were fed with a healthy control diet (CD) or fat and sugar-enriched FPC diet since weaning (3 weeks old). At 3 months of age, FPC diet-fed mice were assigned into two subgroups, i.e., with or without inulin (Inu) supplementation for 5 months. All three groups of mice were euthanized when they were 8 months old. **a** Liver morphology and H&E staining of the liver sections (scale bar represents 50 μm), **b** steatosis score, liver weight, liver-to-body weight ratio, serum ALT, and serum ALP. Data expressed as mean ± SD. *n* = 4 per group. *p < 0.05, **p < 0.01, ***p < 0.001

FPC diet intake for 7 months caused steatosis (Fig. 1a). FPC diet-fed mice had macrovesicular fat with grade 3 steatosis, and inulin-fed mice had improved fat scores (Fig. 1b). In addition, inulin-supplemented mice had normal liver weights and liver-to-body weight ratios (Fig. 1b). Moreover, FPC diet-fed mice had elevated ALT and ALP, and inulin supplementation reversed these changes (Fig. 1b).

### Inulin improves hepatic metabolism in FPC diet-fed mice

**Fig. 2:**
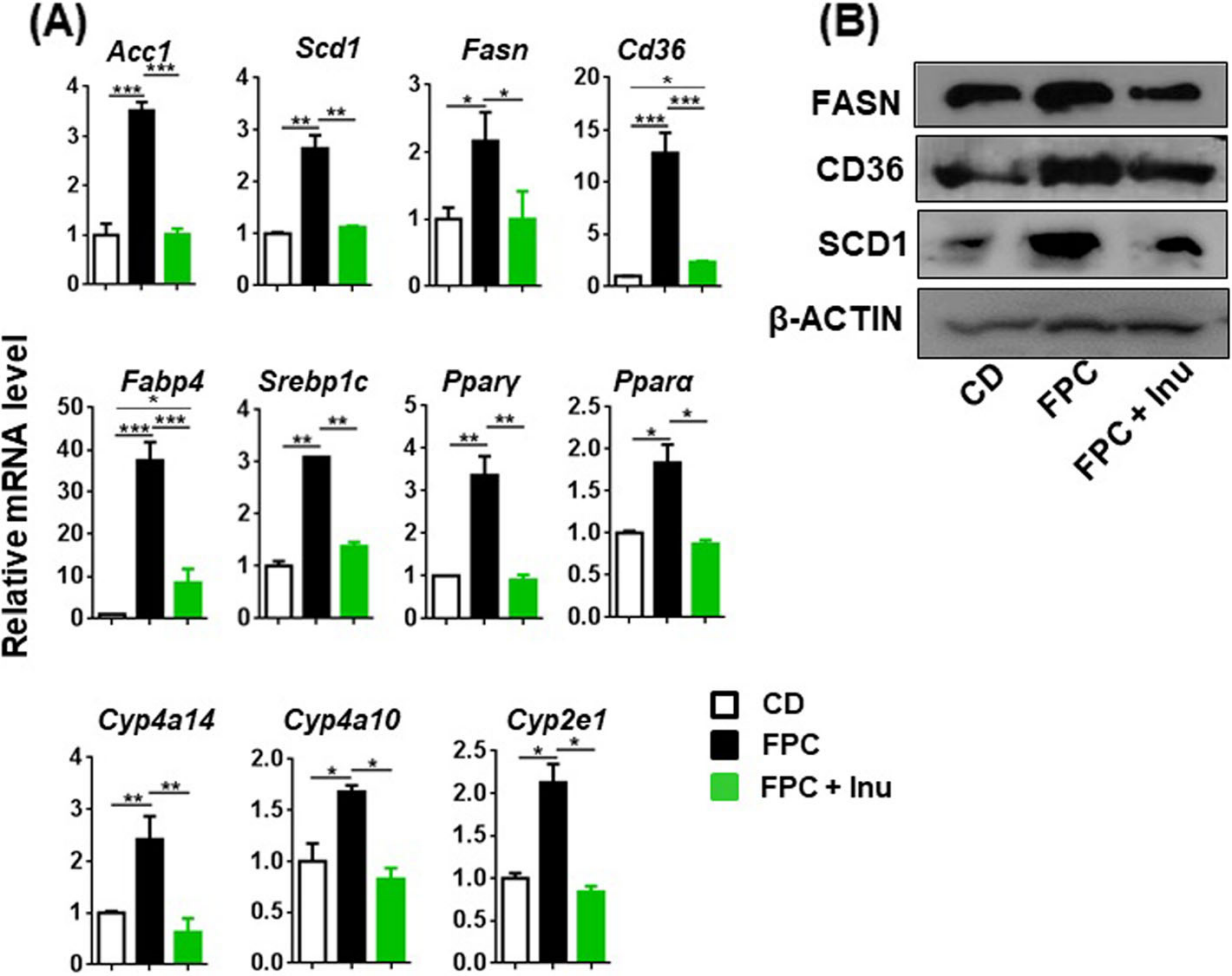
Inulin improves hepatic lipid metabolism. Mice were treated based on the methods described under figure legend 1. **a** Hepatic mRNA levels were quantified by qPCR and **b** hepatic protein levels by Western blot. Representative blots were shown. Data expressed as mean ± SD. *n* = 4 per group. *p < 0.05, **p < 0.01, ***p < 0.001

FPC diet intake increased the expression of genes involved in fatty acid synthesis, uptake, and mobilization as evidenced by increased mRNA levels of Acetyl-CoA carboxylase 1 (*Acc1*), Stearoyl-CoA desaturase-1 (*Scd1*), Fatty acid synthase (*Fasn),* Cluster of differentiation 36 *(Cd36)*, Fatty Acid Binding Protein 4 (*Fabp4*), and Sterol regulatory element-binding protein 1 (*Srebp1c*) as well as genes implicated in fatty acid oxidation such as Peroxisome proliferator-activated receptor α (*Pparα*), PPARα-regulated *Cyp4a14* and *Cyp4a10*, and *Cyp2e1*. In contrast, inulin supplementation prevented these changes in FPC diet-fed mice (Fig. 2a). Similarly, FPC diet increased protein levels of FASN, CD36, and SCD1, which were all reduced by inulin. Representative Western blots are shown in Fig. 2b.

### Inulin prevents FPC diet-induced inflammatory signaling

**Fig. 3:**
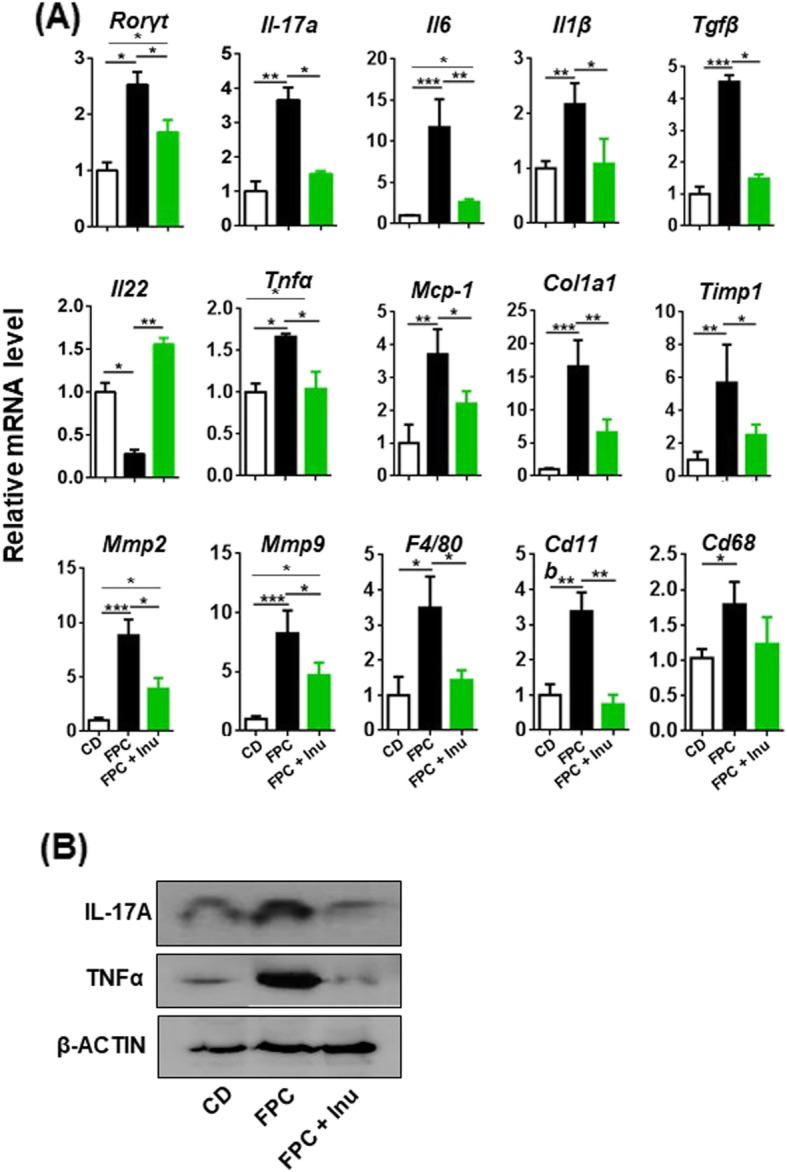
The effect of FPC diet and inulin supplementation on hepatic inflammatory signaling. Mice were treated based on the methods described under figure legend 1. **a** Hepatic mRNA levels were quantified by qPCR and **b** hepatic protein levels by Western blot. Representative blots were shown. Data expressed as mean ± SD. *n* = 4 per group. *p < 0.05, **p < 0.01, ***p < 0.001

IL-17A is implicated in the development of chronic inflammatory diseases in the brain, skin, and liver as well as liver cancer [31–34]. FPC diet-fed mice had increased mRNA level of *Rorγt*, a transcriptional factor for *Il-17* expression, accompanied by elevated hepatic protein levels of IL-17A and TNFα as well as mRNA levels of *Il6*, *Il1β,* and *Tgfβ*, which generate Th17 cells; inulin supplementation prevented these changes (Fig. 3a, b). However, IL10 cytokine family member *Il22* displayed opposite trends in response to FPC diet intake and inulin supplementation (Fig. 3a). Other inflammatory and fibrosis-related genes such as *Tnfα*, *Mcp-1*, *Col1a1*, *Timp1*, *Mmp2*, and *Mmp9* as well as macrophage or neutrophil genes such as *F4/80*, *Cd11b*, and *Cd68* were induced by FPC diet intake and reduced by inulin (Fig. 3a).

### Inulin mitigates FPC-reduced hippocampal LTP and neuroplasticity

**Fig. 4:**
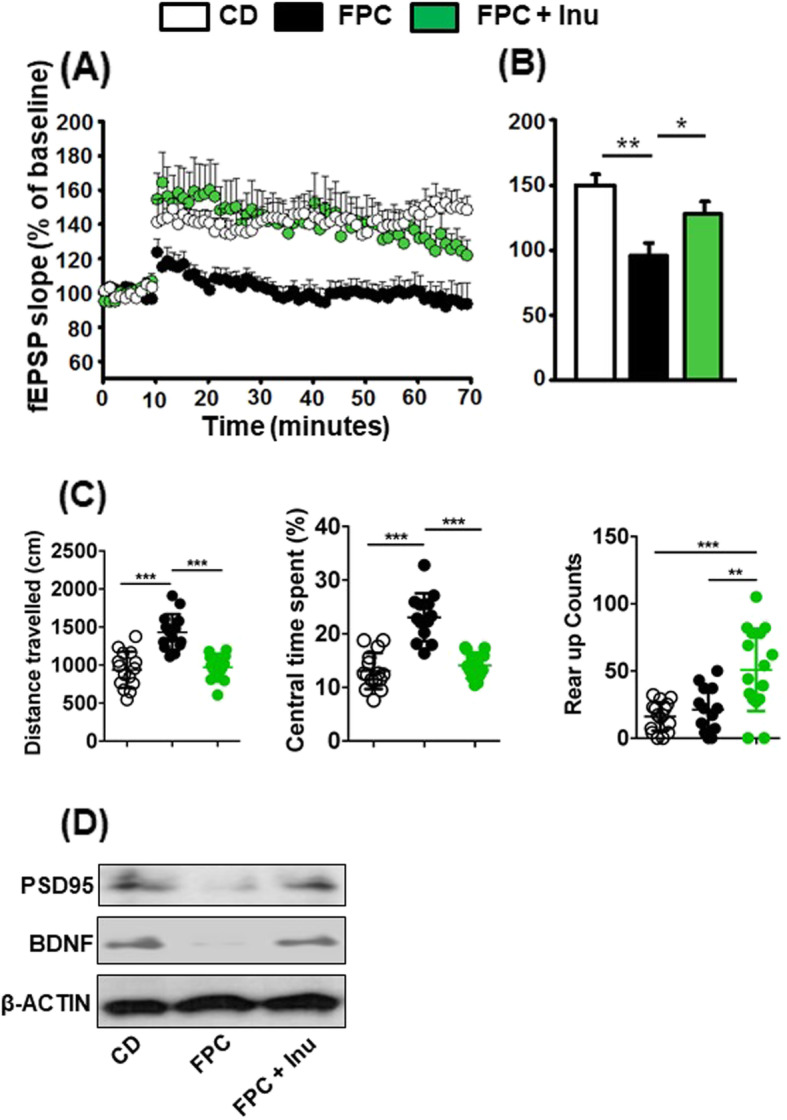
FPC diet-induced synaptic impairment is ameliorated by inulin supplementation. **a** Scatter plot showing high-frequency stimulation-induced LTP, and **b** a bar graph showing LTP calculated by averaging the change in fEPSP slope apparent between 50 and 60 min after high frequency stimulation. All data are presented as the percentage change in fEPSP slope means ± SEM from baseline, **c** Open field behavior study, **d** Western blot data reveal the protein level in brain homogenate. Data expressed as mean ± SD. (*n* = 3 for A-B and *n* = 13–16 for C, and n = 4 for D). *p < 0.05, **p < 0.01

FPC diet-fed mice had reduced LTP at Schaffer collateral-CA1 synapses compared with that of CD-fed mice, and inulin-supplemented mice recovered such a reduction (Fig. 4a, b). In addition, open field behavior study revealed that FPC diet intake increased travel distance and central time spending, and inulin supplementation prevented such increases accompanied by increased rearing (Fig. 4c). Furthermore, FPC diet-fed mice had reduced postsynaptic density-95 (PSD-95), a potent regulator of synaptic strength, as well as brain derived neurotrophic factor (BDNF); however, inulin-supplementation prevented these negative effects caused by FPC intake (Fig. 4d) and improved synaptic plasticity.

### Inulin reverses FPC diet-induced microglia activation

**Fig. 5:**
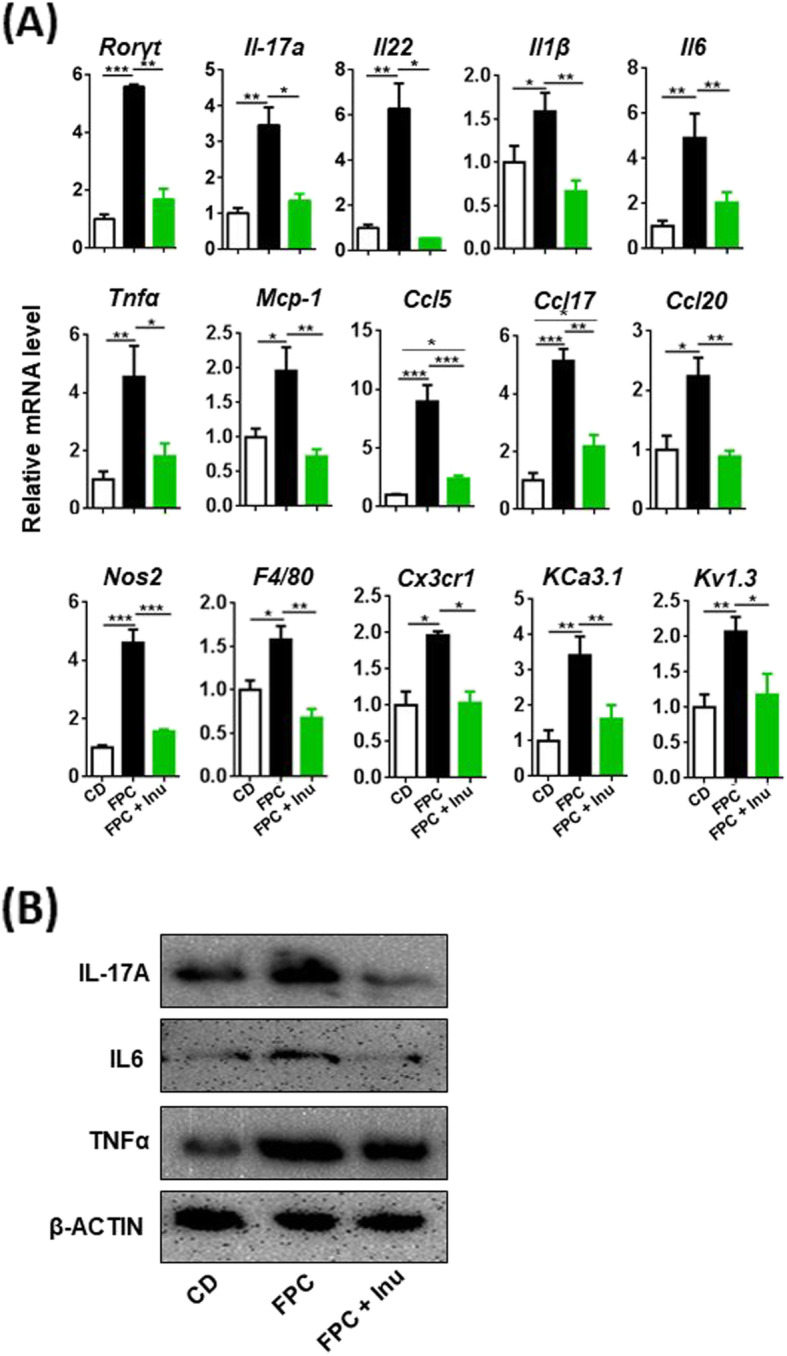
The effects of diet and inulin supplementation on brain inflammation. Mice were treated based on the methods described under figure legend 1. **a** Microglia mRNA level and **b** brain protein level representative image was shown. Data expressed as mean ± SD. *n* = 4 per group. *p < 0.05, **p < 0.01, ***p < 0.001

Microglia have pivotal roles in the inflammatory process in various neurodegenerative conditions; however, little is known about the effect of inulin on microglia activation. In this study, FPC diet-fed mice had elevated mRNA levels of *Rorγt*, *Il-17a,* as well as *Il22* and increased protein levels of inflammatory marker IL-17A, IL6, and TNFα. (Fig. 5a, b). We showed that FPC diet intake increased the expressions of proinflammatory modulators *Il1β*, *Il6*, *Tnfα*, and *Nos2* in the brain, and such inductions were reversed by inulin supplementation (Fig. 5a). Similar expression trends were noted for genes implicated in inflammation or markers for macrophage such as *Mcp-1*, *Ccl5*, *Ccl17*, *Ccl20*, and *Nos2* as well as *F4/80* and *Cx3cr1* (Fig. 5a). The mRNA levels of microglia calcium-activated potassium and voltage-dependent potassium channels *Kca3.1* and *Kv1.2*, which are involved in microglial activation and inflammation, were also elevated in FPC diet-fed mice and reversed by inulin supplementation (Fig. 5a). Together, inulin prevented FPC diet-induced neuroinflammation and microglia activation.

### FPC and inulin supplementation modulate intestinal microbiota

**Fig. 6:**
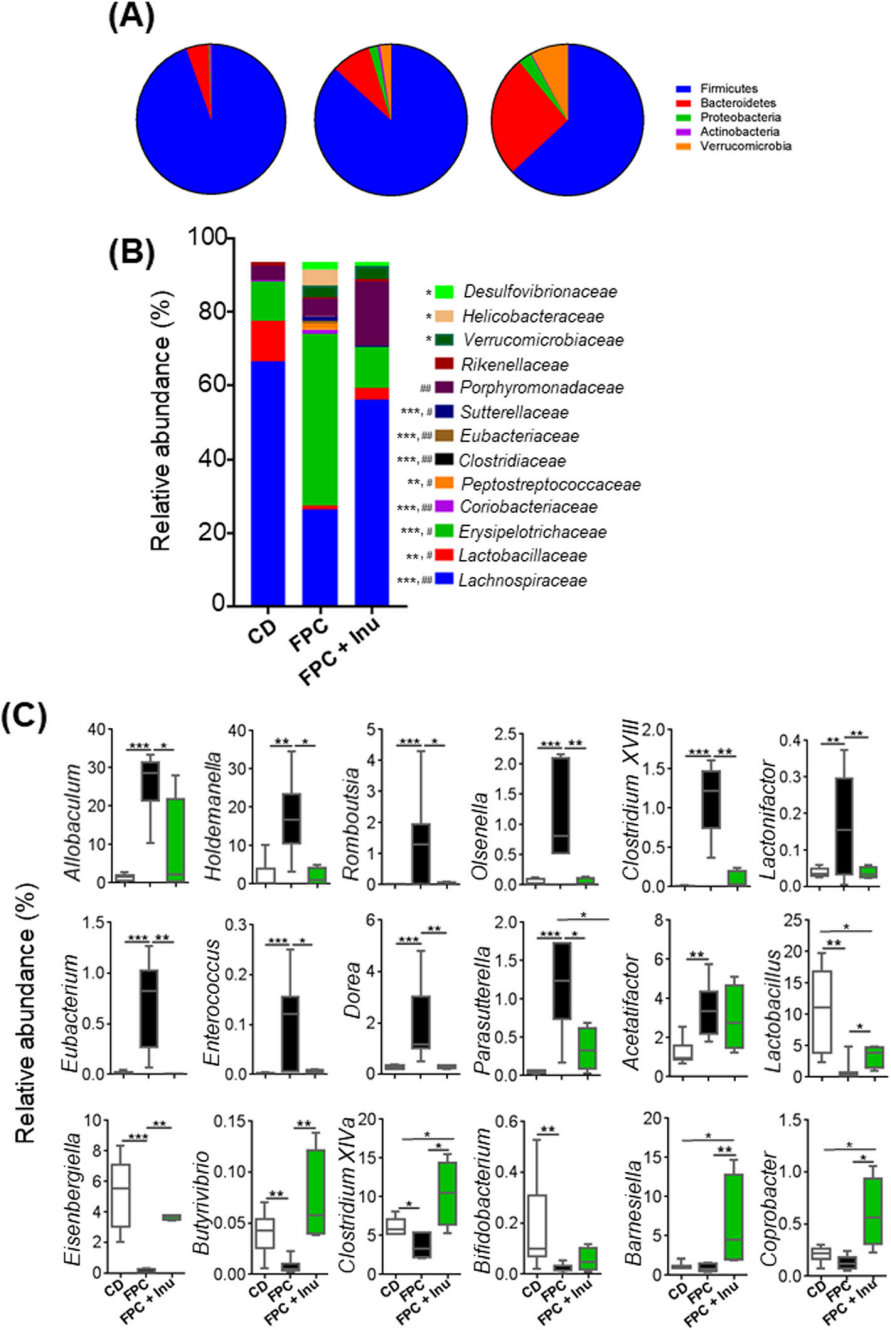
The effects of diet and inulin supplementation on the gut microbiota. Mice were treated based on the methods described under figure legend 1. Relative abundance of cecal microbiota of **a** phylum, **b** family (*, WT vs. FPC; #, FPC vs. FPC + Inulin), and **c** genus levels. Data are expressed as means ± SD (*n* = 8 for CD-fed mice, *n* = 7 for FPC-fed mice, and *n* = 4 for inulin-supplemented FPC-fed mice). *p < 0.05, **p < 0.01, ***p < 0.001

Firmicutes and Bacteroidetes are the most abundant gut bacterial phyla. FPC diet-fed mice had reduced Firmicutes while inulin-supplemented mice had increased Bacteroidetes (Fig. 6a). FPC diet intake resulted in increased Proteobacteria, Actinobacteria, and Verrucomicrobia, and inulin intake only reduced the abundance of Actinobacteria (Fig. 6a).

The reduction of Firmicutes in FPC diet-fed mice is a direct result of the reduction in the families *Lachnospiraceae* and *Lactobacillaceae*, which promote gut health and fight inflammation; however, inulin supplementation increased their abundances (Fig. 6b) and reduced inflammation. In contrast, *Erysipelotrichaceae*, which is involved in cholesterol metabolism, as well as *Peptostreptococcaceae, Clostridiaceae*, and *Eubacteriaceae* genera under Firmicutes were enriched by FPC diet, however inulin reduced them (Fig. 6b). Moreover, inulin-fed mice had an increased family under Bacteroidetes, i.e., *Porphyromonadaceae*, which protects the gut from infection in mouse models [35] (Fig. 6b). The family *Helicobacteraceae* under Proteobacteria, which is implicated in gastrointestinal inflammation [36], increased in FPC diet-fed mice, and inulin supplementation reduced it (Fig. 6b). Additionally, increased Proteobacteria in FPC diet-fed mice could be attributed to increased *Sutterellaceae*, which is implicated in gut inflammation as well as autism and Down syndrome [37, 38]. Under Actinobacteria phylum, *Coriobacteriaceae*, which has a role in cholesterol metabolism, increased with FPC diet intake and reduced with inulin supplementation (Fig. 6b).

At the genus level, FPC diet-fed mice had increased levels of *Allobaculum* and *Holdemanella* (*Erysipelotrichaceae* family), *Romboutsia* (*Peptostreptococcaceae* family), *Romboutsia* (*Peptostreptococcaceae* family), *Olsenella* and *Clostridium XVIII* (*Coriobacteriaceae* family), *Lactonifactor* (*Clostridiaceae* family)*, Eubacterium* (*Eubacteriaceae* family)*, Enterococcus* (*Enterococcaceae* family)*,* Parasutterella (Sutterellaceae family), and *Dorea* and *Acetatifactor* (*Lachnospiraceae* family); inulin supplementation resulted in reductions in these genus except for *Acetatifactor* (Fig. 6c). In contrast, FPC diet reduced *Lactobacillus* (*Lactobacillaceae* family), *Eisenbergiella* (*Lachnospiraceae* family), *Butyrivibrio* (*Lachnospiraceae* family), *Clostridium* XIVa *(Clostridiaceae* family), and *Bifidobacterium* (*Bifidobacteriaceae* family), and inulin supplementation resulted in their increases except for *Bifidobacterium*. Under the family *Porphyromonadaceae*, inulin enriched Barnesiella and *Coprobacter* (Fig. 6c).

### Inulin ameliorates diet-induced dysregulated BA signaling

**Fig. 7:**
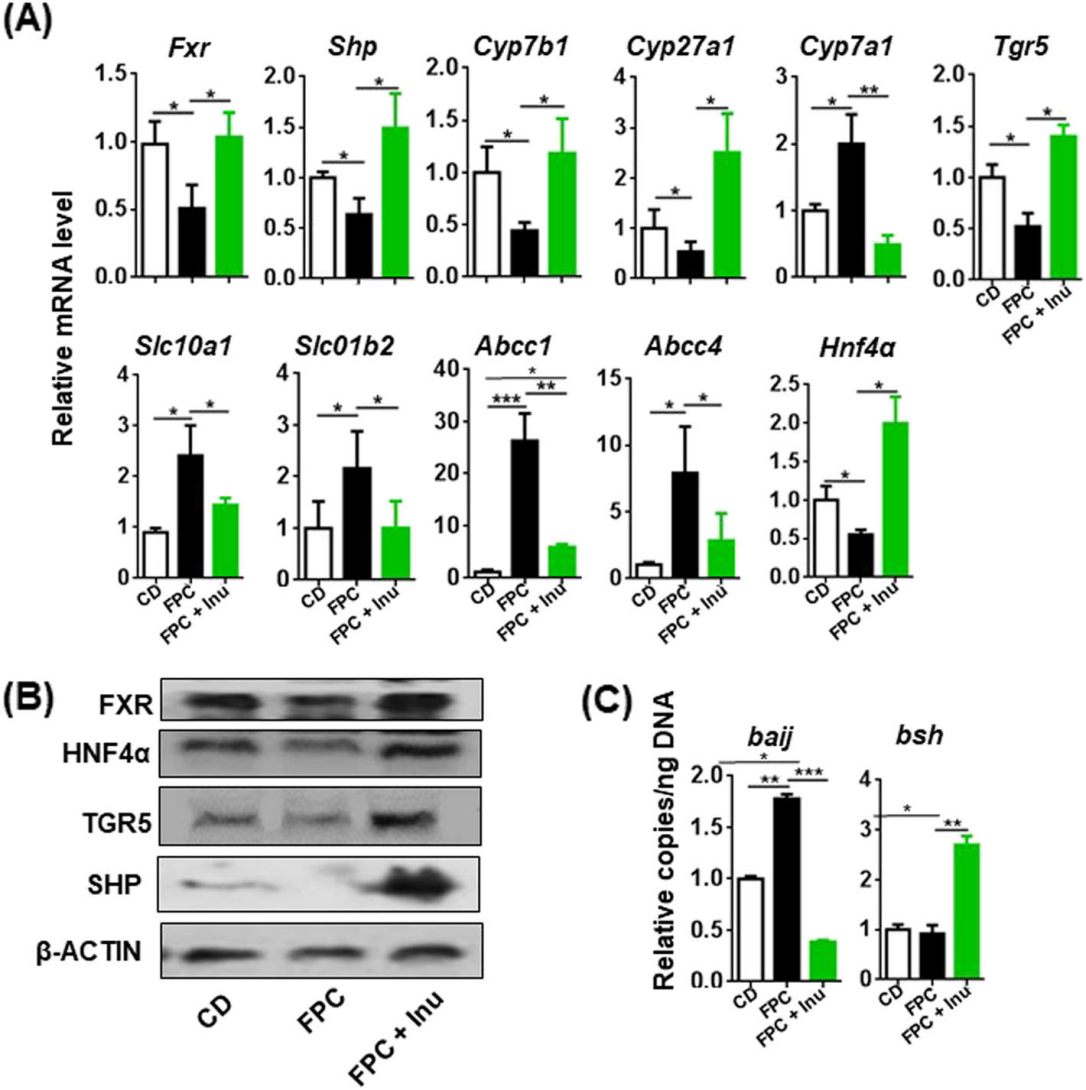
The effect of diet and inulin on expression of genes implicated in bile acid homeostasis. Mice were treated based on the methods described under figure legend 1. **a** Relative level of hepatic mRNA was quantified by qPCR, **b** hepatic protein expression level by Western blot, and **c** the copy number of fecal bacteria genes that generate BAs was quantified by qRT-PCR. (*baiJ*, bile acid inducible operon gene of 7α-hydroxylase; *bsh*, bile salt hydrolase), Data expressed as mean ± SD. *n* = 4 per group. *p < 0.05, **p < 0.01, ***p < 0.001

BA synthesis is jointly regulated by host and gut microbes. Dysregulated BA synthesis is implicated in hepatic inflammation and neurological diseases [24, 39]. We therefore studied the expression of genes that regulate BA homeostasis. FPC diet-fed mice had reduced mRNA and protein levels of FXR and its target gene small heterodimer partner (*Shp*) as well as its encoded protein, which were reversed by inulin supplementation (Fig. 7a, b). In consistency, FXR-regulated genes that are responsible for BA synthesis such as hepatic *Cyp7b1* and *Cyp27a1* had the same expression patterns (Fig. 7a). Additionally, the level of *Cyp7a1*, a rate-limiting enzyme for BA synthesis, was increased in the steatotic livers but reduced by inulin supplementation (Fig. 7a). Moreover, the mRNA and protein levels of hepatic TGR5, another FXR target gene, were reduced in FPC diet-fed mouse livers but increased with inulin supplementation (Fig. 7a, b). Further, BA uptake genes *Slc10a1* and *Slc01b2* as well as transporter genes *Abbc1* and *Abbc4* were induced in FPC diet-fed mice, and inulin supplementation reversed these inductions, suggesting normalization of BA homeostasis (Fig. 7a). Lastly, hepatic HNF4α was reduced by FPC diet intake and increased by inulin supplementation, which likely contributes to normalizing the hepatic transcriptional machinery (Fig. 7a, b). Together, diet-induced metabolic overburden inactivates FXR signaling, and inulin normalizes those changes.

The copy number of bacterial genes that produce BAs was also quantified using cecal DNA. FPC diet-fed mice had increased BA inducible operon *J* (*baiJ*), a gene involved in BA 7α-dehydroxylation, and inulin supplementation reduced it (Fig. 7c). However, the abundance of bile salt hydrolase gene (*bsh*), which hydrolyzes conjugated BAs into free BAs, was unchanged by FPC diet intake but was increased by inulin (Fig. 7c).

TGR5 is abundantly expressed in the macrophage [40, 41]. We thus studied TGR5-regulated signaling in freshly isolated microglia. In consistency with the data generated from the digestive tract, FPC diet-fed mice had reduced *Tgr5* as well as TGR5-regulated *Nos1* and *Dio2* (Figure S2). The expressions of FXR and its target gene *Shp* were also reduced in the microglia of FPC-fed mice, and inulin supplementation increased their expression levels (Figure S2). Thus, diet and inulin have an impact on BA-regulated signaling at the systemic level.

### The effect of FPC diet and inulin on gut metabolites

Metabolomics profiling was performed using cecum content by GC-TOF-MS, which identified 273 known metabolites. As per sparse partial-least-squares discriminant analysis (sPLS-DA), metabolites had different clusters (Figure S3). Pathway analysis revealed that the most significant difference between CD- and FPC diet-fed mice occurred in lipid metabolism that includes steroid hormone, BA, steroid, and fatty acid biosynthesis (Supplementary Table 2, Figure S3). However, inulin supplementation markedly changed sucrose metabolism in addition to amino acid and carbohydrate metabolism pathways, arginine, proline, butanoate, and starch. The top 15 most significantly changed pathways in response to FPC diet intake and inulin supplementation are listed in Supplementary Table 2 and Figure S3.

**Fig. 8:**
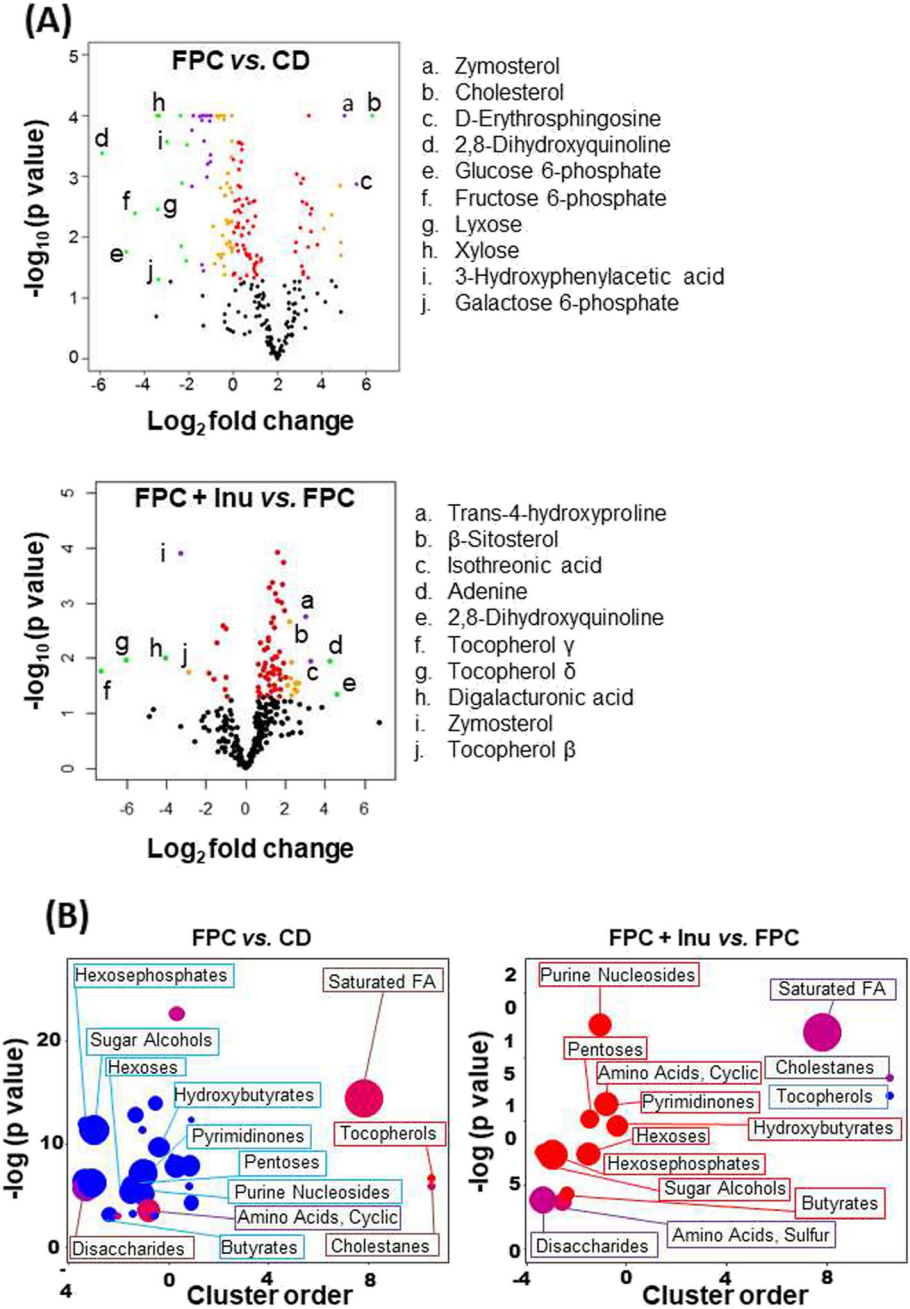
Untargeted metabolomics study of cecal content. Mice were treated based on the methods described under figure legend 1. Volcano plots represent the cecal metabolomics profile between **a** FPC diet vs. CD and FPC diet + Inulin vs. FPC diet. The orange, purple, and green color represent the fold changes of 2, 3, and 4, respectively with the *p*-value < 0.05. **b** ChemRICH metabolite set enrichment statistics plot. The node color shows increased (red) or decreased (blue) metabolite sets in FPC diet vs. CD and FPC diet +Inulin vs. FPC diet. Only enrichment clusters are shown that are significantly different at *p* < 0.05 (The node sizes represent the total number of metabolites in each cluster set)

Fold changes in the cecal metabolites are shown in the volcano plots (Fig. 8a). Zymosterol and 2, 8-dihydroxyquinoline (2, 8-DHQ) are commonly found in both FPC vs. CD and FPC + inulin vs. FPC plots. Zymosterol, the precursor of cholesterol, was increased in FPC diet-fed mice but was reduced by inulin intake. In contrast, 2, 8-DHQ was markedly reduced in FPC diet-fed mice, and inulin supplementation increased it (Fig. 8a). Additionally, glucose 6-phosphate, fructose 6-phosphate, galactose 6-phosphate, xylose, and lyxose had reduced concentrations in FPC diet-fed mice (Fig. 8a). 3-Hydroxyphenylacetic acid, a metabolite of the flavonoid rutin which protects against glucose intolerance and obesity, was decreased due to FPC diet intake. In contrast, tocopherols (β, γ, δ) and digalacturonic acid were increased by FPC diet intake (Fig. 8a). Supplementation of inulin increased trans-4-hydroxyproline, β-sitosterol, isothreonic acid, and adenine (Fig. 8a).

Chemical similarity enrichment analysis shows that FPC diet-fed mice had reduced hexose phosphates, purine nucleosides, pyrimidinones, and sugar alcohols, which were increased by inulin supplementation (Fig. 8b). In contrast, cholestanes, a cholesterol metabolite, was increased in FPC diet-fed mice, and inulin supplementation reduced it (Fig. 8b). In addition, butyrate, a major SCFA produced by fermentation of inulin, was decreased by FPC diet intake and increased by inulin supplementation (Fig. 8b).

### The relationships between metabolites and the gut microbiota

**Fig. 9:**
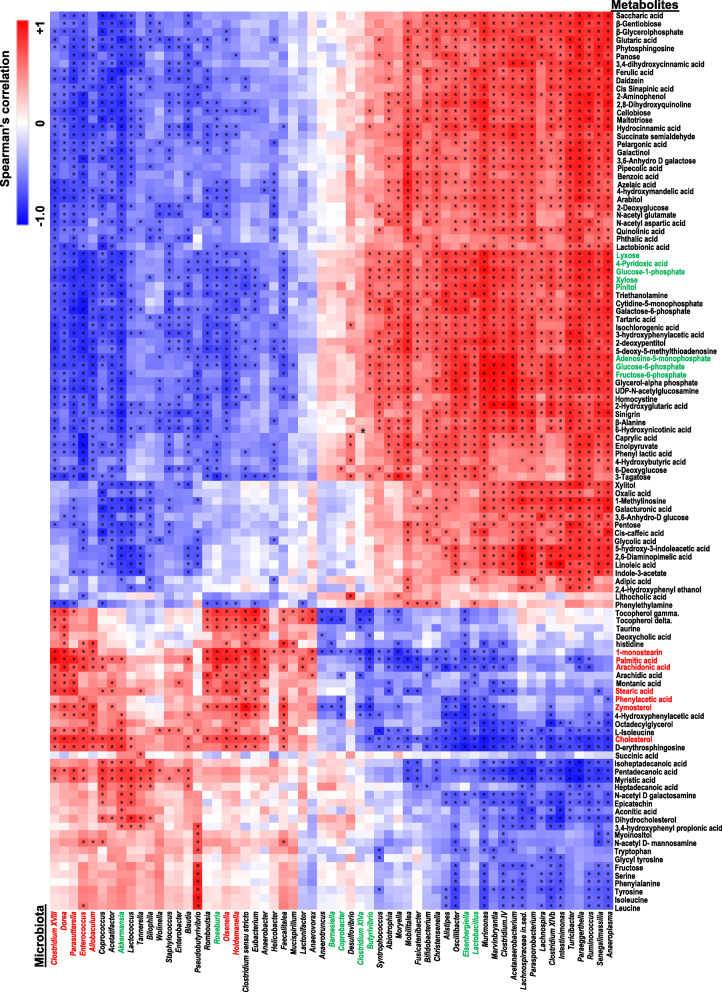
The relationships between the gut microbes and metabolites affect by diet and inulin supplementation. Mice were treated based on the methods described under figure legend 1. A heat map generated by Spearman’s correlation analysis reveals the relationships between the abundance of 59 bacterial genus and 104 metabolites that are significantly changed by diet or inulin supplementation. The spearman correlation data were generated using R programming (*n* = 8 for CD-fed mice, *n* = 7 for FPC-fed mice, and *n* = 4 for inulin-supplemented FPC-fed mice)

Among the 273 identified metabolites, 104 had significant changes in their scaled intensity due to FPC diet intake or inulin supplementation. These metabolites and 59 bacterial genera found in the mouse cecum were subjected to Spearman’s correlation analysis (Fig. 9).

Genus *Allobaculum*, which was increased in FPC diet-fed mice, was positively associated with cholesterol and zymosterol, but negatively correlated with sugar alcohols (xylose, and lyxose); glycolysis pathway metabolites (glucose-6-phosphate and fructose-6-phosphate); and pinitol, which has a role in regulating insulin function (Fig. 9). In addition, genus *Holdemanella* was positively correlated with the scaled intensity of cecum fatty acids stearic acid and palmitic acid as well as fatty acid byproduct monostearin (Fig. 9). Genus *Barnesiella*, *Coprobacter*, *Clostridium XIVa*, and *Butyrivibrio*, which are negatively correlated with tocopherol, zymosterol, and monostearin, had increased abundances in inulin-fed mice (Fig. 9). Moreover, *Lactobacillus* and *Eisenbergiella* genus, which also increased due to inulin supplementation, were negatively associated with cholesterol, zymosterol, and fatty acids D-erythrosphingosine, palmitic acid, stearic acid, as well as arachidonic acid (Fig. 9). These fatty acids are clustered together and positively associated with the bacteria that were enriched in FPC diet-fed mice (Fig. 9).

## Discussion

Our data revealed the detrimental effects of a WD, which simultaneously affects brain and liver health. Gut microbiota and their metabolites, which includes BAs, contributed to such an impact. It was also revealed through our data that inulin can reverse the pathological and molecular changes even while mice continue to consume a WD. Inulin reduced lipogenic gene such as *Fasn*, *Cd36*, *Srebp1c*, and *Pparγ*. It also induced the expression of genes responsible for fatty acid oxidation and mobilization. Those metabolic benefits likely contribute to improved liver and brain phenotypes.

Recent study has shown that inulin induced cholestatic liver cancer in TLR5 knockout mice [19]. However, such a negative impact was not noted in the current study although long-term inulin supplementation was employed. These conflicting findings could be attributed to differences in animal models, diet, or environment, emphasizing the importance of precision nutritional supplementation [20]. However, both studies revealed the significant roles of BAs and the gut microbiota in liver disease development. Moreover, the current study suggests that hepatic metabolic status is an indicator for cognitive function or vice versa. Such a linkage is likely mediated through gut microbiota-driven inflammatory signaling.

IL-17, a cytokine produced by T helper 17 (Th17) cells plays a significant role in hepatic inflammation by inducing neutrophil infiltration and fatty acid release [42]. Increased IL-17A is also found in mouse models of Alzheimer’s diseases and hepatic fibrosis [43, 44]. Moreover, our recent publication shows that short-term feeding of another WD leads to T helper type 1−/T helper type 17-biased skin inflammation before significant body weight gain is noted [31]. WD intake for 4 weeks promotes mild dermatitis and accumulation of IL-17A-producing γδ T cells in the skin. Furthermore, supplementation with cholestyramine, a BA sequestrant, can prevent skin inflammation along with a reduction in the infiltration of γδ T cells and the expression of proinflammatory mediators [31]. The differentiation of Th17 cells relies on a variety of cytokines, and transcriptional factors such as RORγt and TGFβ in synergy with IL6 are critical for generating Th17 cells [45]. Additionally, BAs have been found to be able to modulate RORγt signaling [46]. The simultaneous induction of microglial and hepatic *Rorγt* and *Il-17a* along with other inflammatory cytokines in FPC diet-fed mice, as well as their reductions in inulin-supplemented mice, suggest the significance of IL-17A in diet-associated inflammatory signaling at the systemic level.

A reduction in PSD-95 is associated with postsynaptic degeneration, altered synaptic plasticity, psychiatric diseases, dementia, and Alzheimer’s disease pathology [47]. BDNF is a vital neurotransmitter modulator required for memory and learning [48]. Our data revealed that FPC diet intake reduced LTP, BDNF, and PSD-5; however, inulin supplementation increased all of them. WD intake and obesity are associated with increased anxiety in humans and mice [49, 50]. Here, we showed that inulin supplementation can modulate anxiety as well as exploratory behavior, revealing its beneficial effects. Together, the inflammatory signaling regulated by diet or inulin supplementation not only affects disease development but also has a significant impact on neuroplasticity and behavior.

Regarding the gut microbiota, FPC diet increased *Erysipelotrichaceae* and *Coriobacteriaceae*, which are associated with the dyslipidemia phenotypes found in obese individuals and animal models [51, 52]. In contrast, inulin supplementation reduced *Erysipelotrichaceae* and *Coriobacteriaceae*, suggesting their roles in host lipid metabolism. It is likely that a diet rich in fat and cholesterol increased the abundances of *Allobaculum*, *Holdemanella*, and *Olsenella*, which obese mice and NAFLD patients also have increased abundance [53–55]. Our data revealed that inulin could prevent these changes along with reducing gut cholesterol and zymosterol. In consistency, APP/PS1 mouse model of Alzheimer’s disease also had decreased *Allobaculum* [56]. Probiotic *Lactobacillus* has beneficial effects in improving insulin sensitivity as well as memory and cognition [57, 58]. Our data showed that FPC diet intake reduced *Lactobacillus* and butyrate producing *Butyrivibrio*, and inulin supplementation increased their abundances. *Barnesiella* is an effective immunomodulator, which prohibits the colonization of pathogenic antibiotic-resistant bacteria in the gut [59]; *Barnesiella* was also increased in inulin-supplemented mice. Together, fermentable fiber inulin markedly shifted the gut microbiota as well as the metabolic phenotype, thereby generating health benefits.

BAs are generated by both host and microbial enzymes via metabolizing cholesterol. BA receptor FXR is pivotal for regulating metabolism and inflammation. In consistency, FXR knockout mice spontaneously develop NASH and liver cancer [60]. Our data suggest that FPC diet reduced FXR and FXR-regulated downstream effects. Additionally, HNF4α, a master regulator for hepatic gene expression, is also down regulated in FPC-fed mice. These compromised signaling pathways may account for NAFLD development. However, inulin supplementation restored them. In addition to regulating hepatic metabolism, FXR has a role in altering motor activity, cognitive function, and mood [10]. FXR agonist is currently in clinical trials to treat metabolic liver diseases [61]. Whether FXR agonists can be used to improve neuroplasticity warrants further investigation.

In addition to FXR, systemic inflammation is also accompanied by reduced TGR5 signaling. In consistency, the neuroprotective effect of TGR5 has also been shown in Aβ1–42-induced cognitive impairment mouse models [12]. Additionally, TGR5 activation prohibits hepatic encephalopathy [11, 62]. In the current study, the neuroprotective effect of TGR5 was further supported by the findings that inulin-improved neuroplasticity was accompanied by increased TGR-5-regualted signaling genes such as *Dio2* and *Nos1* in the microglia. Thus, via improving metabolism and reducing inflammation, TGR5 can be a potential target for increasing neuroplasticity as well.

Metabolomics data showed that 2, 8-DHQ, a gut microbe-generated metabolite, was markedly reduced by FPC but increased by inulin. 2, 8-DHQ is a species-specific aryl hydrocarbon receptor agonist and has known benefits in reducing glucose intolerance and fighting obesity [63]. Additionally, glucose 6-phosphate, fructose 6-phosphate, galactose 6-phosphate, and sugar alcohols xylose and lyxose were reduced due to FPC diet intake. Conversely, inulin supplementation increased the abundance of sugar alcohols, which have lower caloric value, clearly indicating increased fermentation. Moreover, inulin increased the production of butyrate, which can be an energy source for gut epithelial cell renewal. ChemRICH analysis showed FPC diet-fed mice had decreased hexose phosphates, purine nucleosides, and pyrimidinones, which were all increased with inulin supplementation. Hexose phosphate molecules are key molecules in the pentose phosphate pathway which generates NADPH used in fatty acid biosynthesis [64]. Moreover, inulin-supplemented mice also had increased trans-4-hydroxyproline, which is implicated in wound healing [65] and β-Sitosterol, which boosts immunity [66]. Furthermore, isothreonic acid, a metabolite of vitamin C, and adenine, known as vitamin B4, were all elevated in the cecum of inulin-fed mice.

Metabolites such as palmitic acid are increased in neurodegenerative diseases such as Parkinson’s disease and Alzheimer’s disease [67]. Certain fatty acids such as monostearin, palmitic acid, arachidonic acid (phospholipids), stearic acid, zymosterol, and D-Erythrosphingosine, which clustered together, were positively correlated with FPC diet-increased microbiota *Dorea*, *Parasutterella*, *Enterococcus*, *Allobaculum*, *Olsenella*, and *Holdemanella* and negatively associated with inulin-increased *Lactobacillus* and *Eisenbergiella*. Thus, gut microbes likely altered the production of neurotransmitter-related metabolites and affected neural function.

## Conclusions

Our results indicate that, chronic consumption of FPC diet negatively impact liver health and cognition. Supplementing fermentable fiber inulin into FPC diet reduced the impact of FPC diet and improved cognitive health. Taken together, diet-associated gut microbes and their derived metabolites through inflammatory signaling such as IL-17A and bile acid receptor signaling concomitantly affect the health of the liver and brain.

## Supplementary Information

**Additional file 1: Table S1.** Primers used for qPCR. **Table S2.** Metabolic Pathways and Function Analysis.

**Additional file 2: Figure S1.** Quantification of western blots showed in Figs. 2b, 3b, 4d, 5b and 7b. (A) Relative protein level of hepatic lipid biosynthesis and metabolism pathway, (B) relative protein level of hepatic inflammation, (C) relative protein level of brain homogenate, (D) relative protein level of brain inflammation, and (E) relative protein level of hepatic bile acid metabolism and synthesis. Data expressed as mean ± SD. *n* = 4 per group. *p < 0.05, **p < 0.01, ***p < 0.001.

**Additional file 3: Figure S2.** The effect of diet and inulin on microglia bile acid signaling. Microglia were freshly isolated to extract RNA. The mRNA levels were quantified by qPCR. Data expressed as mean ± SD. *n* = 4 per group. *p < 0.05, **p < 0.01, ***p < 0.001.

**Additional file 4: Figure S3.** The effect of diet and inulin on metabolomics profile. Untargeted metabolomics profile of cecal sample of CD-fed, FPC-fed, and FPC-fed with inulin supplemented mice. (A) sPLS-DA based analysis of cecal metabolomics, (B) Pathway enrichment analysis of FPC vs. CD fed and FPC + Inu fed vs. FPC fed mice based on small molecule pathway database, (C) Pathway analysis of FPC vs. CD fed and FPC + Inu. fed vs. FPC fed mice based on KEGG database, (D) Heatmap of cecal metabolites clustered with Pearson and Wald statistical analysis (*n* = 8 for CD-fed mice, *n* = 7 for FPC-fed mice, and *n* = 4 for inulin-supplemented FPC-fed mice).

## Data Availability

All data generated or analyzed during this study are included in this article.

## Abbreviations

FPC: Fructose, palmitate, and cholesterol
CD: Control diet
WD: Western diet
H&E: Hematoxylin and eosin
NAFLD: Non-alcoholic fatty liver disease
NASH: Non-alcoholic steatohepatitis
PVDF: Polyvinylidene difluoride
CA: Cornu ammonis
ALT: Alanine transaminase
ALP: Alkaline phosphatase
BA: Bile acid
FXR: Farnesoid x receptor
TGR5: Takeda G protein-coupled receptor 5
LTP: Long-term potentiation
ACC: Acetyl CoA carboxylase
FASN: Fatty acid synthase
fEPSP: Field excitatory postsynaptic potentials
HCC: Hepatocellular carcinoma
TLR: Toll-like receptor
2, 8-DHQ: 2, 8-dihydroxyquinoline
AHR: Aryl hydrocarbon receptor
ACC1: Acetyl-CoA carboxylase 1
SCD1: Stearoyl-CoA desaturase-1
Cd36: Cluster of differentiation 36
Fabp4: Fatty acid binding protein 4
Srebp1c: Sterol regulatory element-binding protein 1
Pparα: Peroxisome proliferator-activated receptor α
Bdnf: Brain derived neurotrophic factor
PSD-95: Postsynaptic density-95
HFD: High-fat diet
SCFA: Short-chain fatty acid
DCA: Deoxycholic acid
