# Supplementary material for: Dysregulated bile acid receptor-mediated signaling and IL-17A induction are implicated in diet-associated hepatic health and cognitive function
Source: Biomark Res. 2020 Nov 6;8:59. doi: 10.1186/s40364-020-00239-8 (PMC7648397; doi:10.1186/s40364-020-00239-8)
Supplement: Supplementary file 1 — Additional file 1: Table S1. Primers used for qPCR. Table S2. Metabolic Pathways and Function Analysis. [file 40364_2020_239_MOESM1_ESM.docx]

**Supplementary table 1**: Primers used for qPCR.

| Primer | Forward | Reverse |
| --- | --- | --- |
| *Acc1* | ATGTCCGCACTGACTGTAACCA | TGCTCCGCACAGATTCTTCA |
| *Scd1* | CGTTCCAGAATGACGTGTACGA | AGGGTCGGCGTGTGTTTC |
| *Fasn* | CCCGGAGTCGCTTGAGTATATT | GGACCGAGTAATGCCATTCAG |
| *Cd36* | TCCAGCCAATGCCTTTGC | TGGAGATTACTTTTTCAGTGCAGAA |
| *Fabp4* | CGTAAATGGGGATTTGGTCA | GCTCTTCACCTTCCTGTCGT |
| *Srebp1c* | TGTGATCTA CTTCTTGTGGCCCGT | AGGCTGCTCAGGTCATGTTGGAAA |
| *Ppar γ* | AGCAGTTTGGCACAGCTAGGTT | TGGCAAGGAATTGTGGTCAGT |
| *Ppar α* | GATTCAGAAGAAGAACCGGAACA | TGCTTTTTCAGATCTTGGCATTC |
| *Cyp4a14* | CAAGACCCTCCAGCATTTCC | GAGCTCCTTGTCCTTCAGATGGT |
| *Cyp4a10* | TCCAGGTTTGCACCAGACTCT | TCCTGGCTCCTCCTGAGAAG |
| *Cyp2e1* | CGCTTCGATTACGATGACAA | GTGTTCCTTGGCTTTTCCAA |
| *Rorγt* | ACCTCCACTGCCAGCTGTGTGCTGTC | CAAGTTCAGGATGCCTGGTTTCCTC |
| *Il-17a* | TCCAGAAGGCCCTCAGACTA | ACACCCACCAGCATCTTCTC |
| *Il6* | GTTGCCTTCTTGGGACTGATG | GGGAGTGGTATCCTCTGTGAAGTCT |
| *Il1β* | AAGATGAAGGGCTGCTTCCA | GTGCTGCTGCGAGATTTGAA |
| *Tgfβ* | GAGCCCGAAGCGGACTACTA | GTTTTCTCATAGATGGCGTTGTTG |
| *Il22* | TTGAGGTGTCCAACTTCCAGCA | AGCCGGACGTCTGTGTTGTTA |
| *Tnfα* | TCGAGTGACAAGCCTGTAG | GTTGGTTGTCTTTGAGATCC |
| *Mcp-1* | GCAGTTAACGCCCCACTCA | CCCAGCCTACTCATTGGGATCA |
| *Col1a1* | CCCGCCGATGTCGCTAT | GCTACGCTGTTCTTGCAGTGAT |
| *Timp1* | CCTGGTCATAAGGGCTAAATTCA | TTAGTCATCTTGATCTTATAACGCTGC |
| *Mmp2* | ACACTGGGACCTGTCACTCC | TGT CAC TGT CCG CCA AAT AA |
| *Mmp9* | CACCTTCACCCGCGTGTAC | GCTCCGCGACACCAAACT |
| *F4/80* | CTTTGGCTATGGGCTTCCAGTC | CTTTGGCTATGGGCTTCCAGTC |
| *Cd11b* | CAATAGCCAGCCTCAGTGC | GAGCCCAGGGGAGAAGTG |
| *Cd68* | CTTCCCACAGGCAGCACAG | AATGATGAGAGGCAGCAAGAGG |
| *Ccl17* | TACCATGAGGTCACTTCAGATGC | GCACTCTCGGCCTACATTGG |
| *Ccl20* | ACTGTTGCCTCTCGTACATACA | ACCCACAATAGCTCTGGAAGG |
| *Nos2* | TGGTGGTGACAAGCACATTT | AAGGCCAAACACAGCATACC |
| *Cx3cr1* | CAGCATCGACCGGTACCTT | GCTGCACTGTCCGGTTGTT |
| *KCa3.1* | AACTGGCATCGGACTCATGGTTCT | AGTCATGAACAGCTGGACCTCCTT |
| *Kv1.3* | TTGTGGCCATCATTCCTTA | CCTGCTGCCCATTACCTTGT |
| *Fxr* | TGCTCACAGCGATCGTCATC | TCTCAGCGTGGTGATGGTTG |
| *Shp* | GGAGTCTTTCTGGAGCCTTG | ATCTGGGTTGAAGAGGATCG |
| *Cyp7b1* | TTGTAGCCCTCTTTCCTCCA | CTTGTTCCGAGTCCAAAAGG |
| *Cyp27a1* | GAGAGTGAATCAGGGGACCA | CCATTTGGGAAGGAAAGTGA |
| *Cyp7a1* | GAGCCCTCAAGCAATGAAAG | TGACCCAGACAGCGCTCTTT |
| *Tgr5* | GTCAGCTCCCTGTTCTTTGC | CAGGAGGCCATAAACTTCCA |
| *Slc10a1* | TATCAGCCCCCTTCAATTTC | GTGAGCCTTGATCTTGCTGA |
| *Slc01b2* | TGGGGCATTAATTGACAGAA | GCTCCACAGCTGGTTACAGA |
| *Abcc1* | GAGGAGACGTGGAAGCTAATGG | GCCAATGGCCTTCATGTAGTT C |
| *Abcc4* | GAAGTATGACCCCGACGACT | GCACTGCACGTGGTAGAAGT |
| *HNF4α* | GTGCTTCCGGGCTGGCATGAA | AGGTGATCTGCTGGGACAGAACC |
| *Nos1* | GCATCCTGGTTGGCCCA | TGTCGCTGTTGCCAAAAACT |
| *Dio2* | GTTGCTTCTGAGCCGCTC | GCTCTGCACTGGCAAAGTC |
| *baiJ* | TCAGGACGTGGAGGCGATCCA | TACRTGATACTGGTAGCTCCA |
| *bsh* | ATGGGCGGACTAGGATTACC | TGCCACTCTCTGTCTGCATC |

**Supplementary Table 2**

Supplementary table 2. Metabolic Pathways and Function Analysis

| Pathway Name | Hits/total compounds | *p* value | FDR | Function |
| --- | --- | --- | --- | --- |
| CD *vs*. FPC | | | | |
| Steroid hormone biosynthesis | 1/72 | 1.79E-06 | 2.6E-05 | Lipid metabolism |
| Primary bile acid biosynthesis | 4/46 | 1.75E-06 | 2.6E-05 | Lipid metabolism |
| Steroid biosynthesis | 4/35 | 1.67E-06 | 2.6E-05 | Lipid metabolism |
| Fatty acid biosynthesis | 5/43 | 0.000399 | 0.002313 | Lipid metabolism |
| Biosynthesis of unsaturated fatty acids | 8/42 | 0.000358 | 0.002308 | Lipid metabolism |
| Fatty acid metabolism | 1/39 | 5.92E-06 | 5.72E-05 | Lipid metabolism |
| Arachidonic acid metabolism | 1/36 | 0.001089 | 0.00574 | Lipid metabolism |
| Fatty acid elongation in mitochondria | 1/27 | 5.92E-06 | 5.72E-05 | Lipid metabolism |
| Glutathione metabolism | 7/26 | 0.00661 | 0.024106 | Metabolism of other amino acids |
| Galactose metabolism | 9/26 | 0.003508 | 0.016956 | Carbohydrate metabolism |
| Glycerolipid metabolism | 3/18 | 0.004534 | 0.020228 | Lipid metabolism |
| Glyoxylate and dicarboxylate metabolism | 4/18 | 0.005807 | 0.024058 | Carbohydrate metabolism |
| Pentose and glucuronate interconversions | 5/16 | 3.41E-05 | 0.000283 | Carbohydrate metabolism |
| Vitamin B6 metabolism | 1/9 | 1.25E-06 | 2.6E-05 | Metabolism of cofactors and vitamins |
| Linoleic acid metabolism | 1/6 | 0.000122 | 0.000888 | Lipid metabolism |
| FPC *vs.* FPC + Inulin | | | | |
| Arginine and proline  metabolism | 16/44 | 0.019586 | 0.066349 | Amino acid metabolism |
| Butanoate metabolism | 7/22 | 0.012198 | 0.064316 | Carbohydrate metabolism |
| Starch and sucrose metabolism | 10/19 | 0.015605 | 0.066349 | Carbohydrate metabolism |
| Histidine metabolism | 4/15 | 0.019383 | 0.066349 | Amino acid metabolism |
| Nicotinate and nicotinamide metabolism | 2/13 | 0.001872 | 0.029248 | Metabolism of cofactors and vitamins |
| Synthesis and degradation of ketone bodies | 1/5 | 0.002234 | 0.029248 | Lipid metabolism |
| D-Glutamine and D-glutamate metabolism | 3/5 | 0.016357 | 0.066349 | Metabolism of other amino acids |
| Nitrogen metabolism | 4/9 | 0.021635 | 0.066349 | Energy metabolism |
| Porphyrin and chlorophyll metabolism | 2/27 | 0. 02164 | 0.066349 | Metabolism of cofactors and vitamins |
| Pentose phosphate pathway | 3/19 | 0.021735 | 0.066349 | Carbohydrate metabolism |
| Alanine, aspartate and glutamate metabolism | 12/24 | 0.02439 | 0.07073 | Amino acid metabolism |
| Purine metabolism | 12/68 | 0.038821 | 0.10722 | Nucleotide metabolism |
