# Supplementary figures and images for: Dysregulated bile acid receptor-mediated signaling and IL-17A induction are implicated in diet-associated hepatic health and cognitive function
Source: Biomark Res. 2020 Nov 6;8:59. doi: 10.1186/s40364-020-00239-8 (PMC7648397; doi:10.1186/s40364-020-00239-8)

Supplementary Figure 1

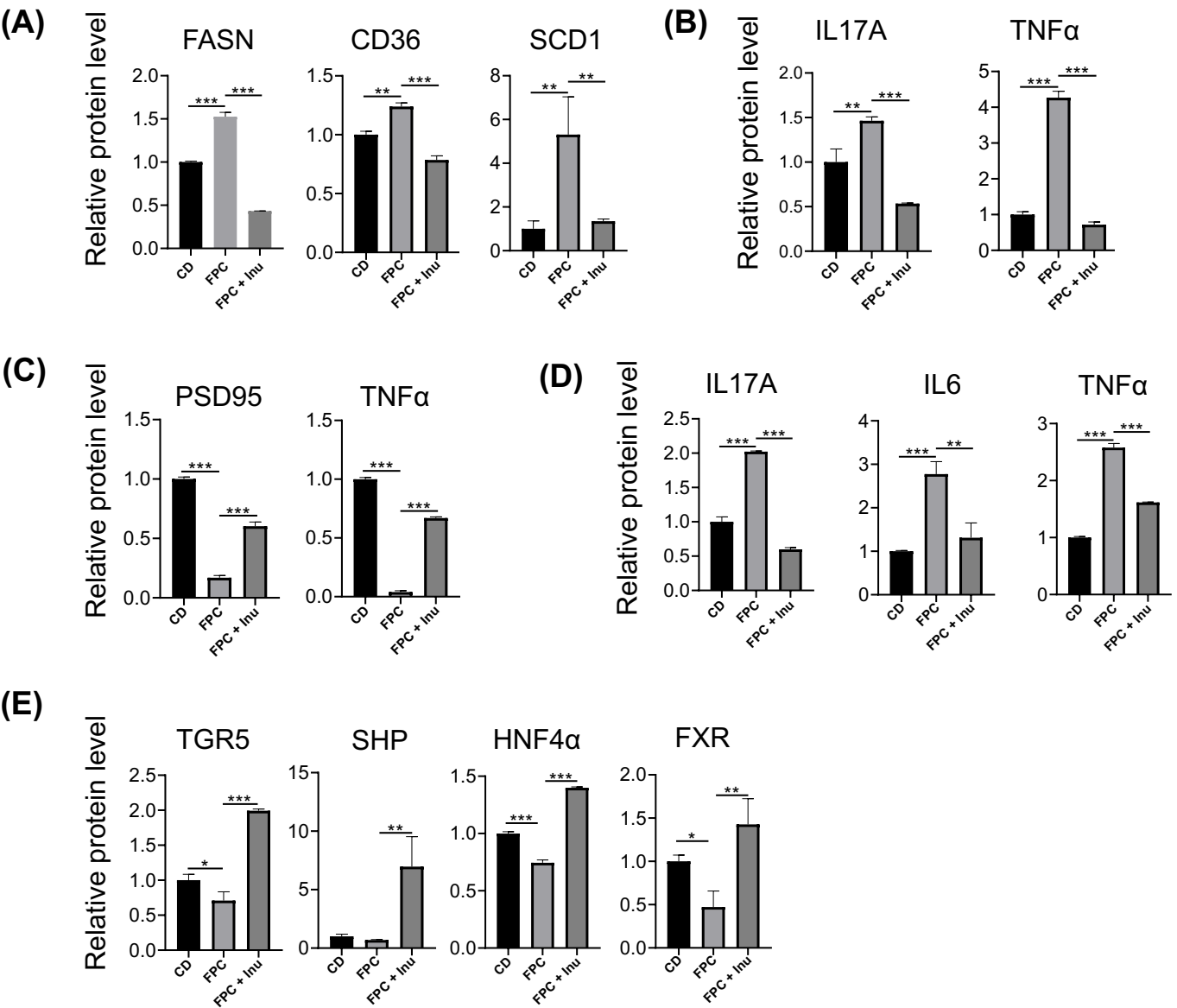

Supplement: Supplementary file 2 — Additional file 2: Figure S1. Quantification of western blots showed in Figs. 2b, 3b, 4d, 5b and 7b. (A) Relative protein level of hepatic lipid biosynthesis and metabolism pathway, (B) relative protein level of hepatic inflammation, (C) relative protein level of brain homogenate, (D) relative protein level of brain inflammation, and (E) relative protein level of hepatic bile acid metabolism and synthesis. Data expressed as mean ± SD. n = 4 per group. *p < 0.05, **p < 0.01, ***p < 0.001. [file 40364_2020_239_MOESM2_ESM.pdf]

Supplementary Figure 2

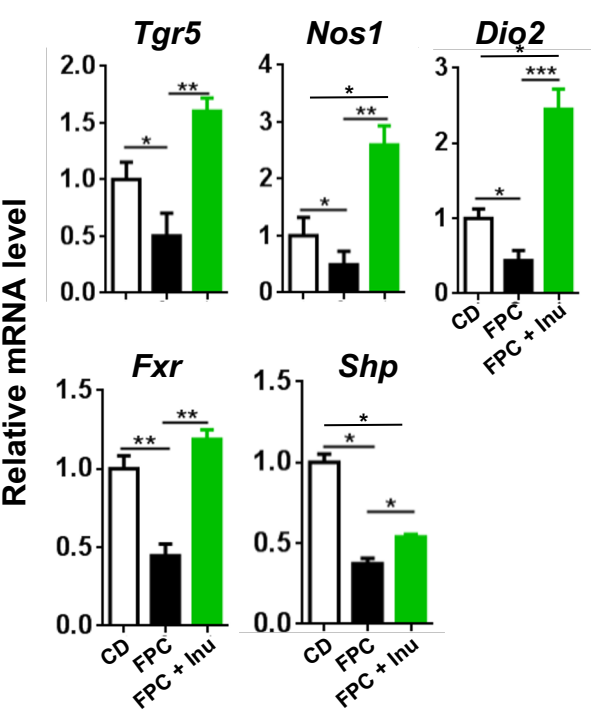

Supplement: Supplementary file 3 — Additional file 3: Figure S2. The effect of diet and inulin on microglia bile acid signaling. Microglia were freshly isolated to extract RNA. The mRNA levels were quantified by qPCR. Data expressed as mean ± SD. n = 4 per group. *p < 0.05, **p < 0.01, ***p < 0.001. [file 40364_2020_239_MOESM3_ESM.pdf]

# Supplementary Figure 3

(A)

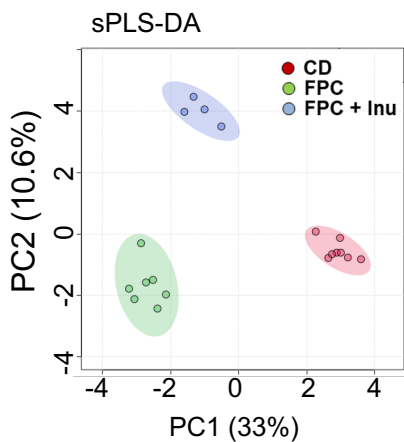

(B)

FPC vs. CD

FPC + Inu vs. FPC

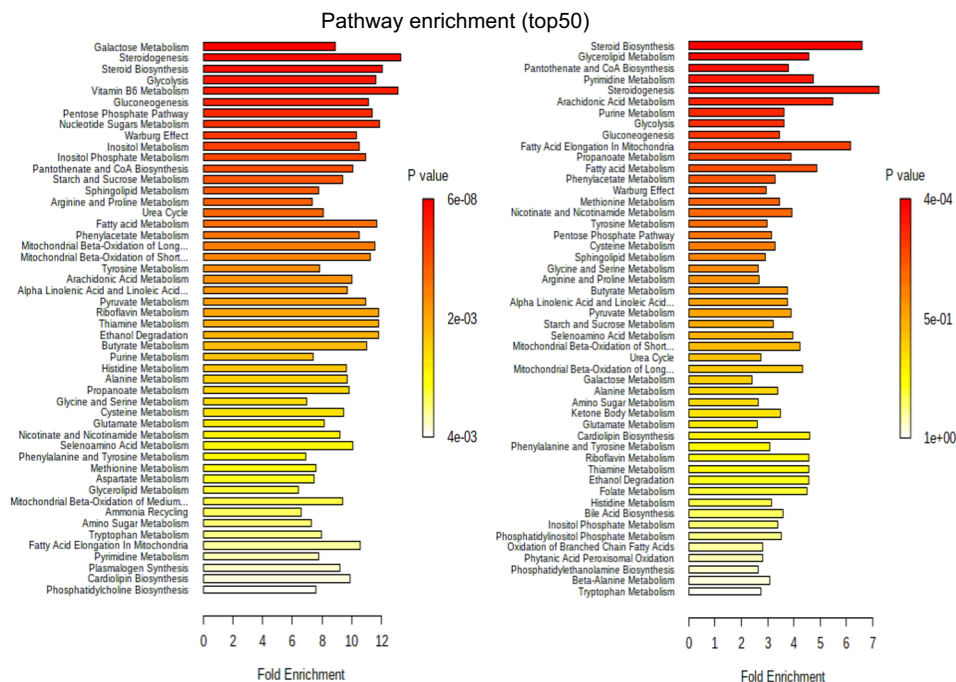

(C)

FPC vs. CD

FPC + Inu vs. CD

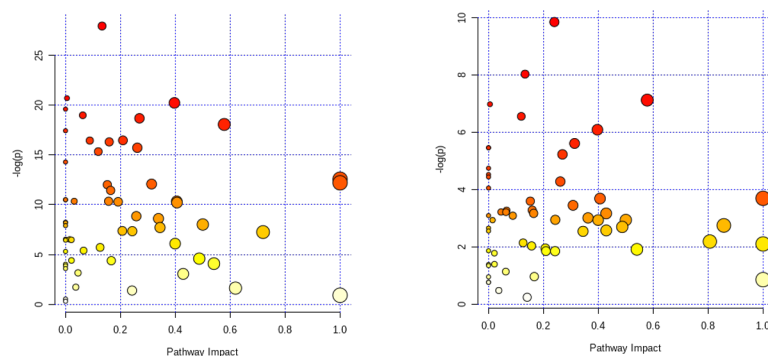

(D)

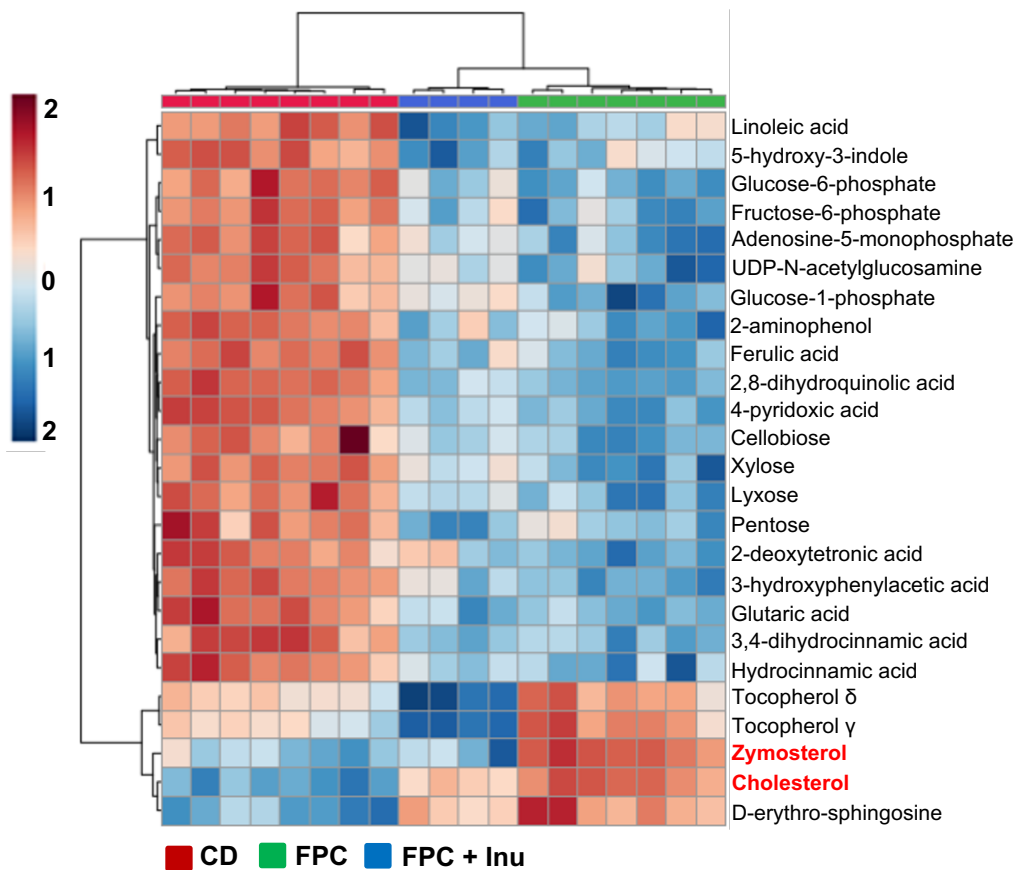

Supplement: Supplementary file 4 — Additional file 4: Figure S3. The effect of diet and inulin on metabolomics profile. Untargeted metabolomics profile of cecal sample of CD-fed, FPC-fed, and FPC-fed with inulin supplemented mice. (A) sPLS-DA based analysis of cecal metabolomics, (B) Pathway enrichment analysis of FPC vs. CD fed and FPC + Inu fed vs. FPC fed mice based on small molecule pathway database, (C) Pathway analysis of FPC vs. CD fed and FPC + Inu. fed vs. FPC fed mice based on KEGG database, (D) Heatmap of cecal metabolites clustered with Pearson and Wald statistical analysis (n = 8 for CD-fed mice, n = 7 for FPC-fed mice, and n = 4 for inulin-supplemented FPC-fed mice). [file 40364_2020_239_MOESM4_ESM.pdf]
